# Discrete element simulation of vibration compaction of slag subgrade

**DOI:** 10.1038/s41598-024-55276-2

**Published:** 2024-02-29

**Authors:** Hu Peng, Chen Jiazhen, Zhang Lejin, Wang Kun, Wang Shuping, Chi Lianyang

**Affiliations:** Shandong Jiaotong University, Jinan, China

**Keywords:** Subgrade engineering, Discrete element model, Slag, Vibration compaction, Civil engineering, Structural materials

## Abstract

In this study, to improve the compaction quality and parameters of slag, discrete element models of irregular rock particles (10–60 mm) and circular soil particles (5 mm) were established based on on-site slag screening results. The motion of the vibratory roller was captured by coupling the roadbed model with the roller model in a simulation in which the roller vibrated and compacted the slag subgrade. The results indicated that (1) the best compaction was achieved when the small particle content was 40%, the medium particle content was 20%, and the large particle content was 40%. (2) When the slag was dominated by small rock particles, the optimum compaction frequency was 28 Hz, and when large rock particles dominated, the optimum compaction frequency was 33 Hz. (3) Rock particles were the primary particles that experienced stress in the vibration compaction, and the compressive force and displacement depended on the particle size. (4) The longitudinal and vertical displacements and rotation angles of the soil and rock particles were examined. The results of this study are conducive for advancing the understanding of slag compaction and improving the working efficiency and compaction quality of rollers.

## Introduction

In road construction, large amounts of rock ballast waste and architectural sediments produced as a result of breaking rocks and filling borrows have caused severe issues, such as environmental pollution and resource waste. As a result, the recycling and utilization of construction waste has garnered considerable attention owing to the increasing awareness of the need for environmental protection.

Slag is the general term that encompasses architectural sediments and residual stone slag waste. In engineering contexts, it is also defined as rock and soil aggregates in which the content of particles with diameters larger than 40 mm is greater than 30% and the maximum particle diameter is no more than 150 mm. Slag is widely applied in subgrade filling to save resources and costs owing to its local availability, extensive sources, high strength, and low settlement deformation. However, the compaction quality of slag is relatively difficult to control because its diameter, grading, moisture content, and gravel strength are not able to be determined^1^. Currently, slag research is primarily conducted through field tests and indoor vibration compaction tests, but knowledge of the compaction mechanism and compaction quality of slag is presently insufficient.

Many laboratory studies on slag compaction parameters have been conducted. For example, through a recently developed flat-plate vibration compaction laboratory test Wang et al.^2^ determined the optimal vibration frequency of a coarse-grained soil filler to be 25 Hz. In addition, Wang et al. and Zhang et al.^3,4^ used X-ray and computed tomography (CT) scanning to explore the effects of rock particle size and content on the stress, strain, and structural changes in soil-rock mixtures. However, laboratory testing requires a significant amount of labor and material resources, and two-dimensional imaging also has certain limitations in studying the properties of mixtures.

As a result, numerical simulations have become a widespread means of studying soil-rock mixtures. For example, Li et al.^5^ showed through finite element simulations that the optimal vibration frequency of a stone-filled roadbed is 28 Hz. However, finite element simulations model materials as continuous and uniform media; consequently, they can only analyze material stress and settlement from a macro perspective^6–8^. In contrast, discrete element simulations can capture the heterogeneity of materials and also analyze the response of materials from both macro and micro perspectives^9–11^. For example, Wersäll et al.^12^ used discrete element simulations to discover that the optimal vibration frequency for soil compaction is 18 Hz. Furthermore, through laboratory tests and three-dimensional discrete element simulations, Ji et al.^13^ recommended a vibration frequency of 25 Hz for a soil-rock mixture. However, the simulation in that study did not account for the driving and rolling of the vibratory roller. Both the vibration frequency and vibration amplitude affect the compaction, and that study only considered the effect of frequency on the compaction of the soil-rock mixture. This is problematic because the physical and mechanical properties of soil-rock mixtures are more complex than those of coarse-grained soil fillers. Compaction parameters are also required to improve the mechanical properties of soil-rock mixtures, and understanding these parameters can contribute to making the material more suitable for use in roads, railways, and other basic projects.

In terms of the particle motion response, Ma et al.^7^ used a finite element simulation to analyze the displacement response regularity that occurs during soil compaction. The results showed that the soil displacement could be divided into rapid growth, uniform, and stable stages; however, this study did not analyze the horizontal displacement. Xiao et al.^14^ and Wang et al.^15^ used SmartRock to study the evolution and regularity of the compaction of graded gravel filler. In addition, Wang et al.^16,17^ and Dan et al.^18^ used SmartRock to study the rotational compaction of aggregate particles in asphalt mixtures, and used the results to explain the motion characteristics of the rotary compaction mechanism. Despite its usefulness, SmartRock has certain measurement limitations in studying particle distributions, settlements, and arrangements. Consequently, few studies have been conducted on the mesoscopic motion response of the compaction of soil-rock mixtures. In summary, research on the influence of the vibration amplitude and its combination mode on the compaction of soil-rock mixtures is still insufficient. Research on the microscopic movement of particles during the vibratory compaction of earth-rock mixtures is also lacking.

In this study, the three-dimensional discrete element software package called Electrical Discrete Element Method (EDEM) and the multi-body dynamics software package Recurdyn were used to jointly simulate slag compaction. The simulation restored the on-site compaction process and reduced the errors in the indoor vertical vibration compaction and two-dimensional discrete element simulations. Through simulations, the influences of various vibration parameters on the porosity of the slag and the number of compactions on the settlement volume were analyzed. In terms of the mesoscopic response, the influence of the compaction parameters on the motion of mixed particles was analyzed, as well as the changes in force, displacement, and rotation of each particle at the edge and center of the subgrade during the compaction process. Further analysis was performed by comparing the microscopic responses of the particles and the effect of the macroscopic compaction. Finally, the simulation results were verified through field tests and compared to previous results. This study proposes reasonable compaction parameters for slag of different grades and provides a basis for understanding the vibration compaction of slag.

The rest of this paper is organized as follows. Section “Multi-body dynamics and discrete element modeling of the subgrade” describes the multi-body dynamics and discrete element modeling of the subgrade, Section “Simulation result” presents the simulation results, Section “Mesoscopic response of particles” discusses the mesoscopic responses of the particles, Section “Further experimental results and analysis” analyzes the results, and Section “Analysis and discussion of results” contains the conclusion of the study.

## Multi-body dynamics and discrete element modeling of the subgrade

### Degree-of-freedom (DOF) model

**Figure 1:**
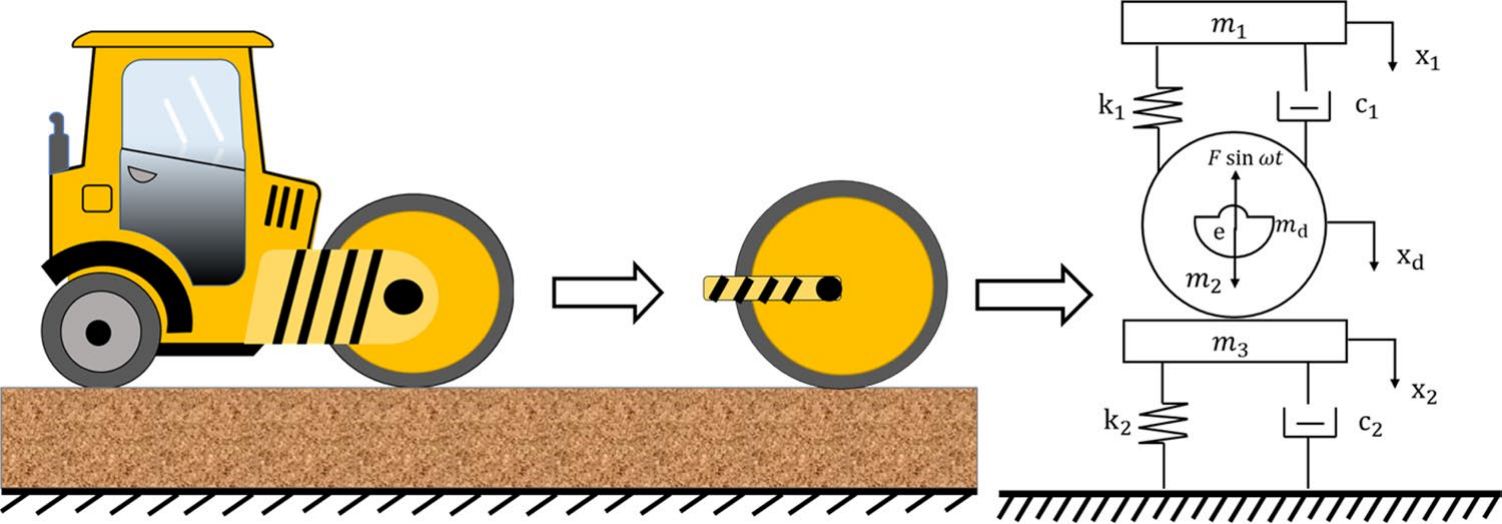
Roller-subgrade coupling model, in which ${m}_{1}$ is the roller frame mass; ${m}_{2}$ is the subgrade mass, ${m}_{3}$ is the steel wheel mass; ${m}_{d}$ is the mass of the eccentric block in the steel wheel; ${x}_{1}$*, *${x}_{2}$*,* ${x}_{d}$ are the vertical displacements of each mass block; *e* is the eccentric distance; ${k}_{1}$ is the stiffness coefficient of the roller, ${c}_{1}$ is the damping coefficient of the roller, ${k}_{2}$ is the subgrade stiffness coefficient, and ${c}_{2}$ is the subgrade damping coefficient.

Xu^19^ pointed out that the fact that, in theory, the vibratory roller filling body is “a dynamic control problem of rolling contact between the rigid cylinder and elastic–plastic bodies under a vibration state”. Therefore, this study assumed that the steel wheel and roller frame were rigid bodies, the steel wheel was always in contact with the subgrade (i.e., no “bounce” occurs), and the model describing the coupling between the roller and roadbed (shown in Fig. 1) was a two-degree-of-freedom (2DOF) model.

**Figure 2:**
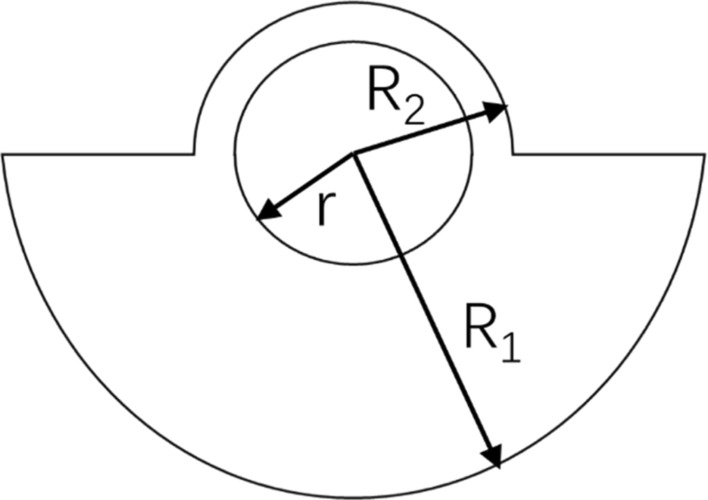
Structure of the eccentric block.

The kinetic equation obtained according to the model and Newton’s second law is given by

1 $$\left\{ {\begin{array}{*{20}l} {m_{1} \ddot{x}_{1} + c_{1} \left( {\dot{x}_{1} - \dot{x}_{2} } \right) + k_{1} \left( {x_{1} - x_{2} } \right) = 0} \hfill \\ {m_{2} \ddot{x}_{2} + \dot{x}_{2} \left( {c_{1} + c_{2} } \right) + x_{2} \left( {k_{1} + k_{2} } \right) - c_{1} \dot{x}_{1} - k_{1} x_{1} = m_{d} e\omega^{2} \sin \omega t} \hfill \\ \end{array} } \right.,$$

where $\omega = 2\pi f$ is the angular speed of the roller eccentric block, $f$ is the roller vibration frequency, and $e$ is the eccentricity of the eccentric block. The latter is structured as a semi-circle, as shown in Fig. 2.

The eccentric distance of the eccentric block is given by

2 $$e = \frac{{4\left( {R_{1}^{3} - R_{2}^{3} } \right)}}{{3\pi \left( {R_{1}^{2} + R_{2}^{2} - 2r^{2} } \right)}},$$

### Multi-body dynamic model

A Xugong XS222J vibratory roller was used as the vibratory compaction machine. The parameters describing the characteristics of the machine are listed in Table 1.

**Table 1:** Main parameters of the vibratory roller.

Parameter	Symbol	Unit	Value
Overall weight	*M*	kg	22,000
Front axle load weight	*m*	kg	11,000
Vibrational frequency	*f*	Hz	25–35
Nominal amplitude	*A*_0_	mm	0.93–1.86
Operational speed	*v*	km/h	2.63

The roller model was constructed by importing a vibrating wheel and an interior eccentric block (the roller’s core working devices) into Recurdyn. The vibration amplitude can be adjusted by changing the angle of the eccentric block, which generates an excitation force via its rotation. The excitation force is the vertical component of the steel wheel generated by the rotation of the eccentric block around the vibration axis. This force acts in accordance with simple harmonic motion, which can be expressed as

3 $$F = m_{3} A_{0} \omega^{2} \sin \omega t = m_{d} e\omega^{2} \sin \omega t,$$

where ${A}_{0}=\frac{e{m}_{d}}{{m}_{3}}$ is the nominal amplitude. The mass of the steel wheel (${m}_{3}$) accounts for 50–60% of the overall weight. The eccentric distance ($e$) can be determined once the design of the eccentric block is completed. Thus, the nominal amplitude can be changed by adjusting the mass of the eccentric block (${m}_{d}$).

**Figure 3:**
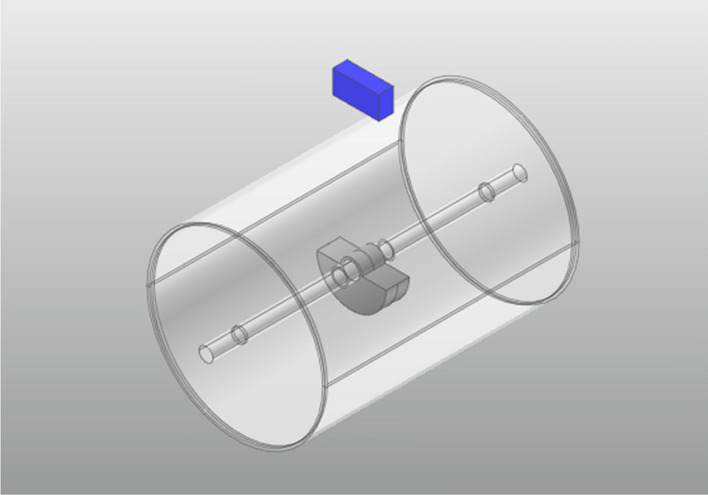
Model of the steel wheel.

To ensure that each component moved correctly, appropriate constraints and motion functions were assigned in Recurdyn. In general, when an object is designed to exhibit two types of motion, it is necessary to build another geometry (called a dummy body) as an assistant. The roller frame was equivalent to a concentrated mass block, and was used as the dummy body to guarantee the movement of the steel wheel. The steel wheel model used in Recurdyn is shown in Fig. 3. The settings of the kinematic pair and driving function imposed on each component are listed in Table 2.

**Table 2:** Settings of the kinematic pair and driving function for each component.

Constraint member	Constraint type	Driving function
Eccentric block—steel wheel	Rotating pair	${m}_{d}e{\omega }^{2}{\text{sin}}\omega t$
Steel wheel—frame	Rotating pair	22.67 rad/s
Frame—ground	Translation pair	2.63 km/h

### Discrete element modeling of roadbed materials

1. Selection of the contact model

**Figure 4:**
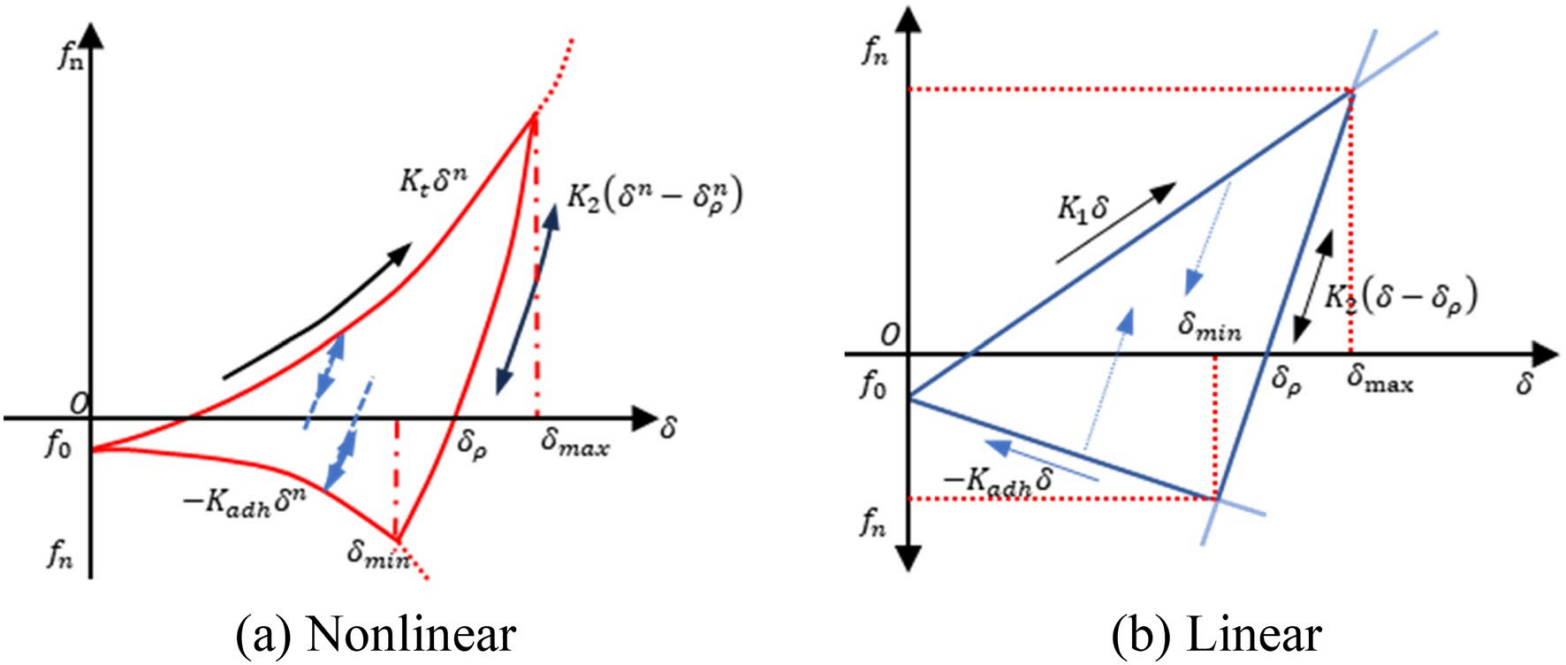
Relationship between the normal contact force and the displacement, as defined in the EEPA contact model^21^, in which *k*_1_ is the original loading stiffness parameter (kN/m), *k*_2_ is the unloading/reloading stiffness parameter (kN/m), *K*_*adh*_ is the normal adhesion force applied according to the adhesion stiffness parameter *f*_*n*_ (N), ${f}_{0}$ is the constant adhesion (N), $\delta$ is the total normal overlap (m), ${\delta }_{max}$ is the maximum normal overlap (m), ${\delta }_{min}$ is the minimum normal overlap (m), ${\delta }_{p}$ is the plastic deformation overlap, and *n* is the nonlinear exponential parameter.

Discrete element modeling (DEM) was initially developed by Cundall and Strack^20^ to analyze granular materials. The discrete element software EDEM can construct various geotechnical models by modeling bulk materials and multiple contact physics and setting different particle sizes and shapes according to the Edinburgh elastoplastic adhesion (EEPA) contact model. This model is based on the integration of a linear spring model, a nonlinear hysteresis spring model, and the Johnson-Kendall-Roberts (JKR) model, and assumes that the tensile strength (adhesive force) increases as the plastic contact area increases, which reflects elastoplastic deformation and adhesion. The EEPA contact model has been widely applied to the interactions between agricultural machinery and soil, as it accurately reflects actual stress characteristics of soil. Therefore, it was selected to model the contact between slags in this study^21–23^. The EEPA model is multifunctional. Thus, the following seven parameters were defined: constant initial bond strength, adhesion energy, contact plasticity ratio (${\gamma }_{p}$), bond branch index (*X*), load branching index (*n*), and shear stiffness (${K}_{tm}$). Figure 4 illustrates the relationship between the normal contact force and the displacement, as defined in the EEPA contact model.

(2) Material parameter setting

**Figure 5:**
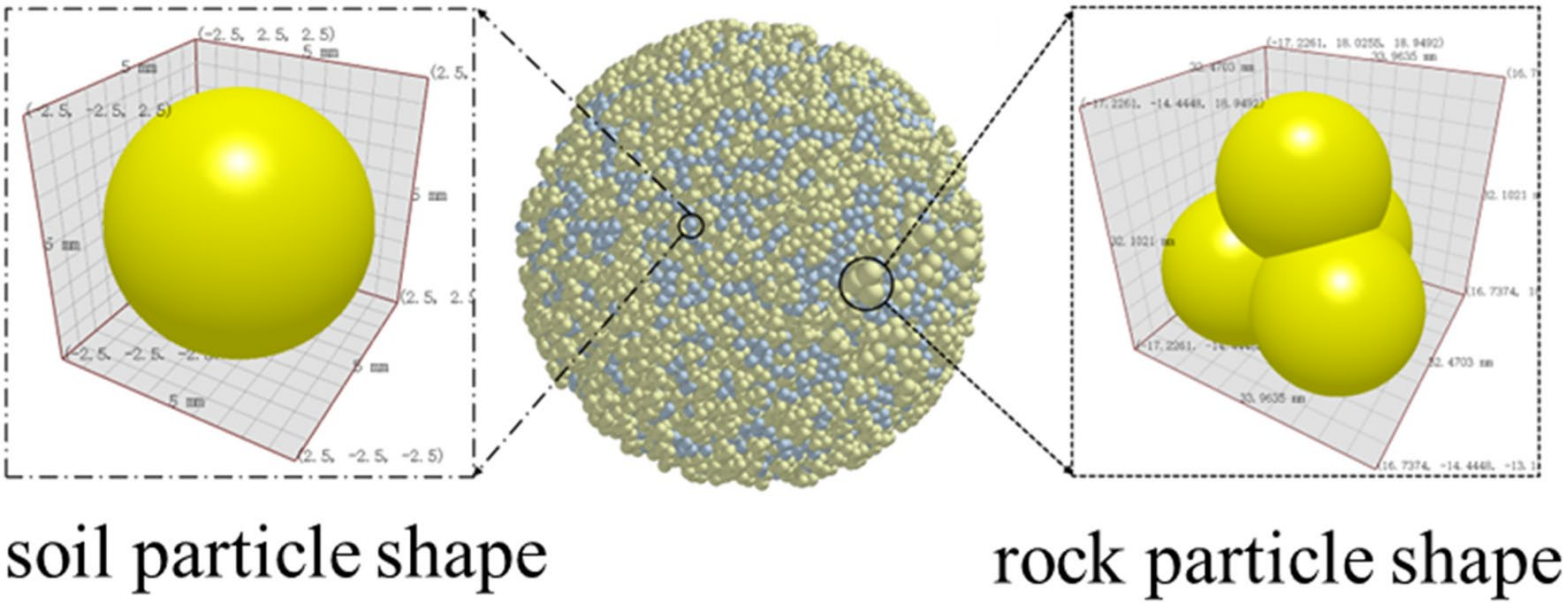
Particle shapes.

According to the literature^22,23^, the size threshold separating soil and stone is 5 mm; that is, slag particles larger than 5 mm are considered rock, and those smaller than 5 mm are considered soil. The eigenvalues and contact parameters of earth-rock materials found in the literature^1,24–28^ are listed in Tables 3 and 4, respectively. The soil particles were single-spherical with a size of 5 mm. Discrete element simulation research has shown that the shape, size, and content of rock particles affect the mechanical properties of soil-rock mixtures^17,29–31^. Accordingly, the rock particle shape was modeled as a tetrahedral spherical particle with a size of 10–60 mm, as shown in Fig. 5. The rock particles were classified into six grades according to particle size, as shown in Table 5. The slag was divided into six gradation types according to the different rock particle sizes, as shown in Table 5. Following the literature^32–34^, the values of the EEPA model’s soil parameters were chosen as follows: constant adhesion ${f}_{0}=0$, contact plasticity ratio ${\gamma }_{p}=0.4$, bond branch index X = 4, load branching index n = 1.5, and shear stiffness ${K}_{tm}=0.4$. The size of the subgrade model was 2 m × 1.2 m × 0.3 m.

**Table 3:** Eigen parameters of materials.

Material type	Poisson’s ratio υ	Density ρ (kg/m^3^)	Shear modulus G (Pa)
Sand	0.38	2130	1.3e+06
Limestone	0.25	2650	2.09e+08
Steel	0.3	7800	7e+10

**Table 4:** Contact parameters between materials.

Material type	Collision coefficient of restitution	Coefficient of static friction	Coefficient of rolling friction
Sand-sand	0.4	0.2	0.1
Sand-steel	0.5	0.6	0.07
Limestone-limestone	0.2	0.8	0.1
Limestone-steel	0.56	0.5	0.07

**Table 5:** Rock particle content of different sizes.

Type	Small rock particle content (10–20 mm) (%)	Medium rock particle content (20–40 mm) (%)	Large rock particle content (40–60 mm) (%)
Gradation 1	10	40	50
Gradation 2	20	40	40
Gradation 3	30	40	30
Gradation 4	40	40	20
Gradation 5	40	20	40
Gradation 6	50	20	30

### EDEM-Recurdyn joint simulation

**Figure 6:**
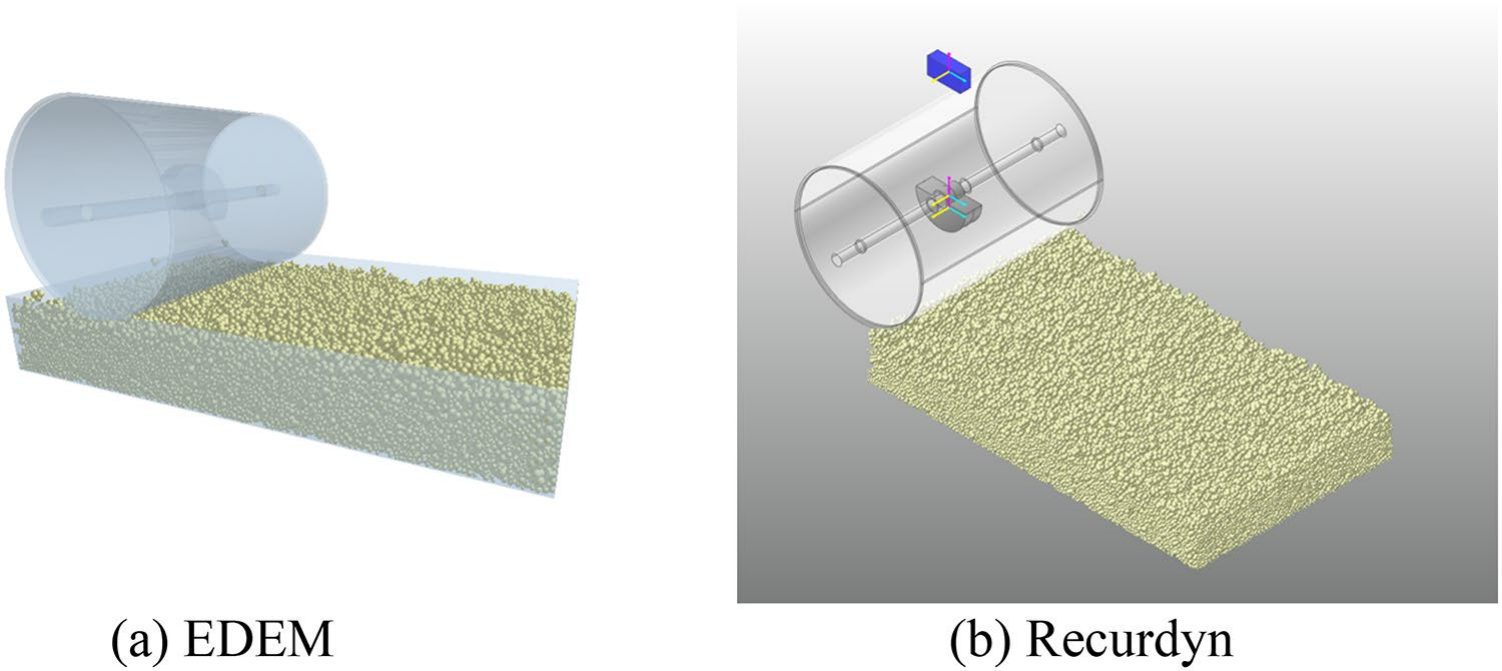
Joint simulation.

In Recurdyn, the combination of the steel wheel and eccentric block were considered a rigid body (wall). The output (a .stl file) was then imported into EDEM for joint simulation. The joint simulation process is illustrated in Fig. 6.

## Simulation result

### Compaction effect section

**Figure 7:**
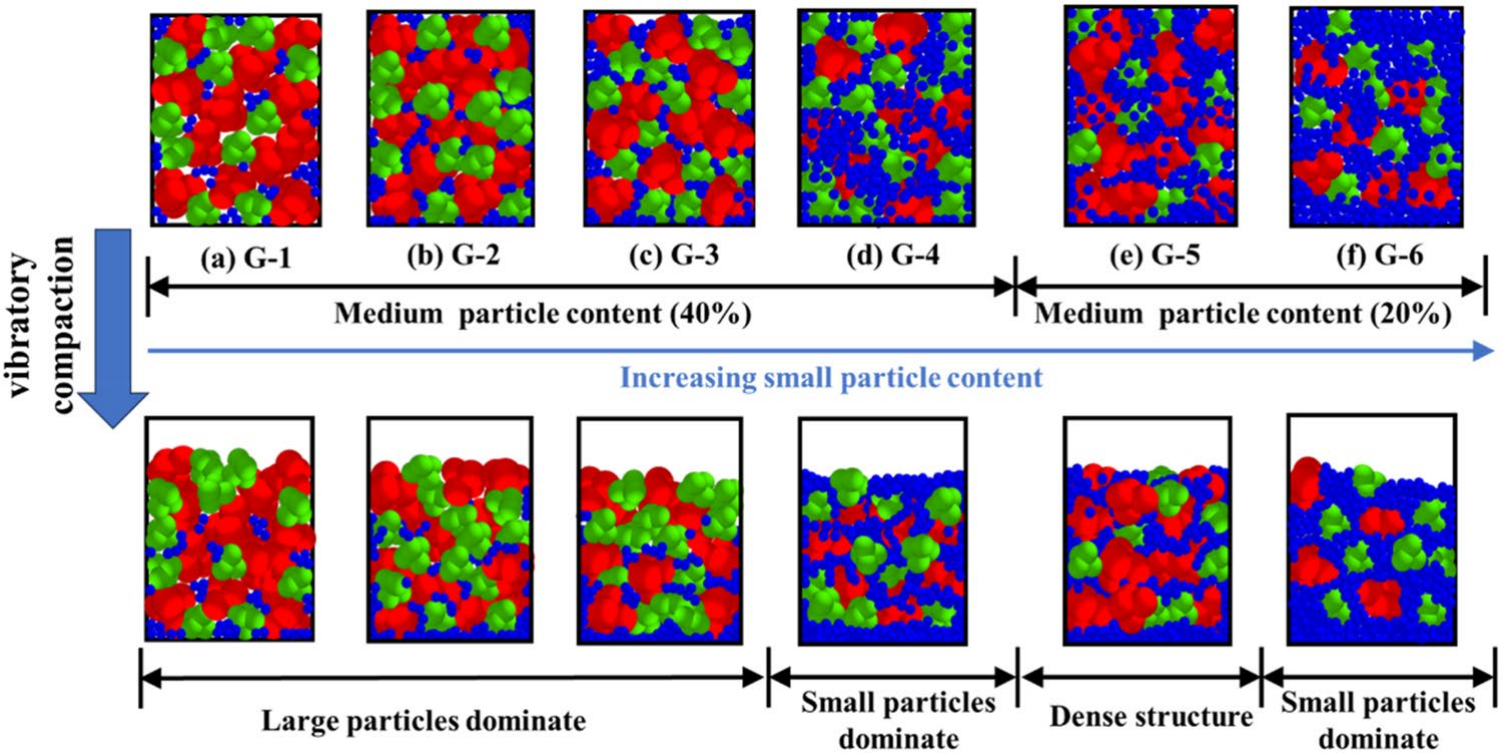
Cross-sectional view of the particles before (top row) and after (bottom row) compaction.

The simulation generated cross-sectional diagrams of the particles with different gradations before and after compaction, which are shown in Fig. 7. As shown in the figure, gradations 1–3 were dominated by large rocks, and the low small particle content resulted in a large number of small gaps between the particles. In gradation 4, some rock particles interlocked with each other to form a skeletal structure; however, there were too many small particles. In gradation 5, small rock particles filled the gaps in the skeleton, which exhibited a dense structure. Gradation 6 was dominated by small rocks, in which large and medium rock particles were suspended.

### Effect of vibration frequency on porosity

**Figure 8:**
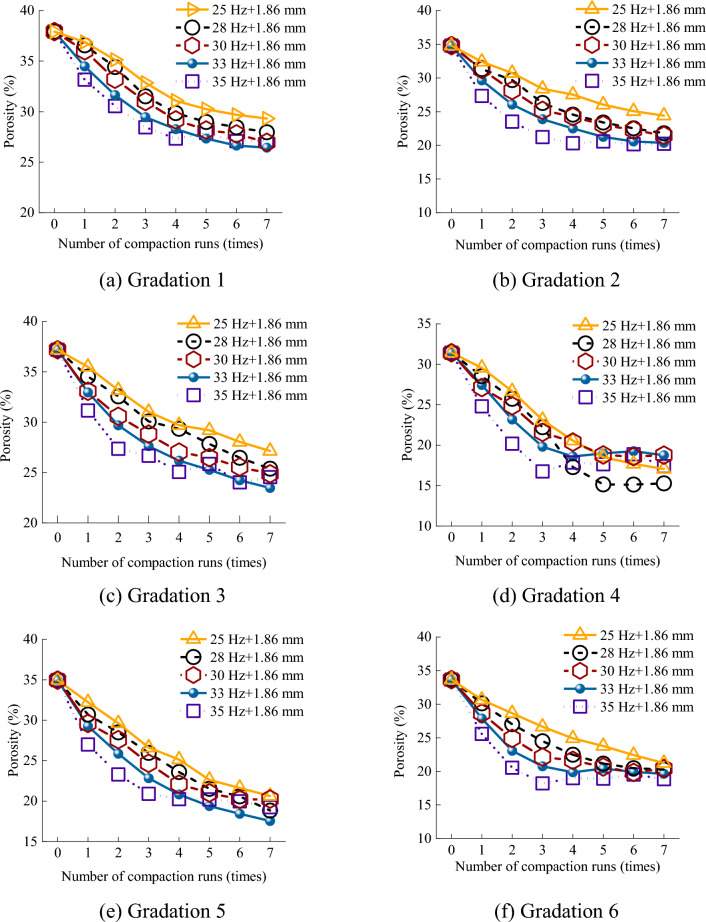
Effect of the vibration frequency and number of compaction passes on the porosity.

In Recurdyn, the frequency can be adjusted by changing the setting of the eccentric block driver function. In EDEM, a 3 × 1 × 3 selection bin group was established and placed in the middle of the subgrade model, and the average value of the porosity in each region was taken as the porosity after each compaction. The simulation results for the porosity as a function of the number of compaction passes for various vibration frequencies are shown in Fig. 8.

Figure 8 shows that the higher the vibration frequency, the faster the porosity decreased. However, the final porosities produced by the different vibration frequencies were not significantly different (Supplementary Information 1). The rates of decrease in the porosity for gradations 1 and 2 were less affected by the frequency, and the porosity was stable after four compaction passes. This occurred because, as shown in Fig. 7a,b, gradations 1 and 2 were dominated by large rock particles, and the friction between the particles was large. At the same time, the small rock particles were insufficient to fill the interparticle gaps. For example, in the first compaction pass, the porosities of gradation 1 at 25 Hz, 28 Hz, and 30 Hz were 36.89%, 36.56%, and 36.02%, respectively, whereas those of gradation 2 were 32.41%, 31.30%, and 31.26%, respectively. The porosities for different vibration frequencies were similar. The porosities of gradation 3 were 35.49%, 34.53%, and 33.08%, and those of gradation 4 were 29.57%, 28.52%, and 27.13%, respectively. However, a very high vibration frequency also caused the mixture to loosen again. For example, at a vibration frequency of 35 Hz, the porosity rebounded in all gradations, and the higher the small particle content, the more obvious the rebound.

When the content of the small rock particles was less than or equal to that of the large rock particles, the best compaction effect was produced at 33 Hz, as shown in Fig. 8a–c. Conversely, the compaction effect produced at 28 Hz was better, as shown in Fig. 8d. Gradations 5 and 6 exhibited a 20% reduction in the medium particle content compared to the other gradations. The change in the optimal vibration frequency still conformed to the pattern of the abovementioned observations, as shown in Fig. 8e,f. This indicates that the optimal vibration frequency depended on the relationship between the small rock particle content and the large rock particle content, with no dependence on the medium rock particle content. In gradations 4 and 5, the small rock particle content was the same, but the medium and large rock particle contents were different. The porosity of gradation 5 did not rebound, whereas that of gradation 4 rebounded by 2.6% and 7.9% at 33 Hz and 35 Hz, respectively. This indicates that large rock particles primarily played a structural support role, whereas medium rock particles primarily acted as bridges between large rocks, increasing the overall stability. In addition, as shown in Fig. 7e, the small and medium rock particles in gradation 5 filled the gaps between the rocks, which is the optimal gradation. Figure 8 shows a comprehensive illustration of the impact of the vibration frequency on the porosity; the maximum difference in the final compaction porosity between 28 Hz, 30 Hz, and 33 Hz was no more than 3.5%. In addition, during the compaction at 28 Hz, the mixture did not rebound, even when there were many small rock particles. Therefore, it is recommended to use a vibration frequency of 28 Hz for compaction at construction sites, as this frequency achieves excellent compaction and reduces mechanical losses.

### Effect of vibration amplitude on porosity

**Figure 9:**
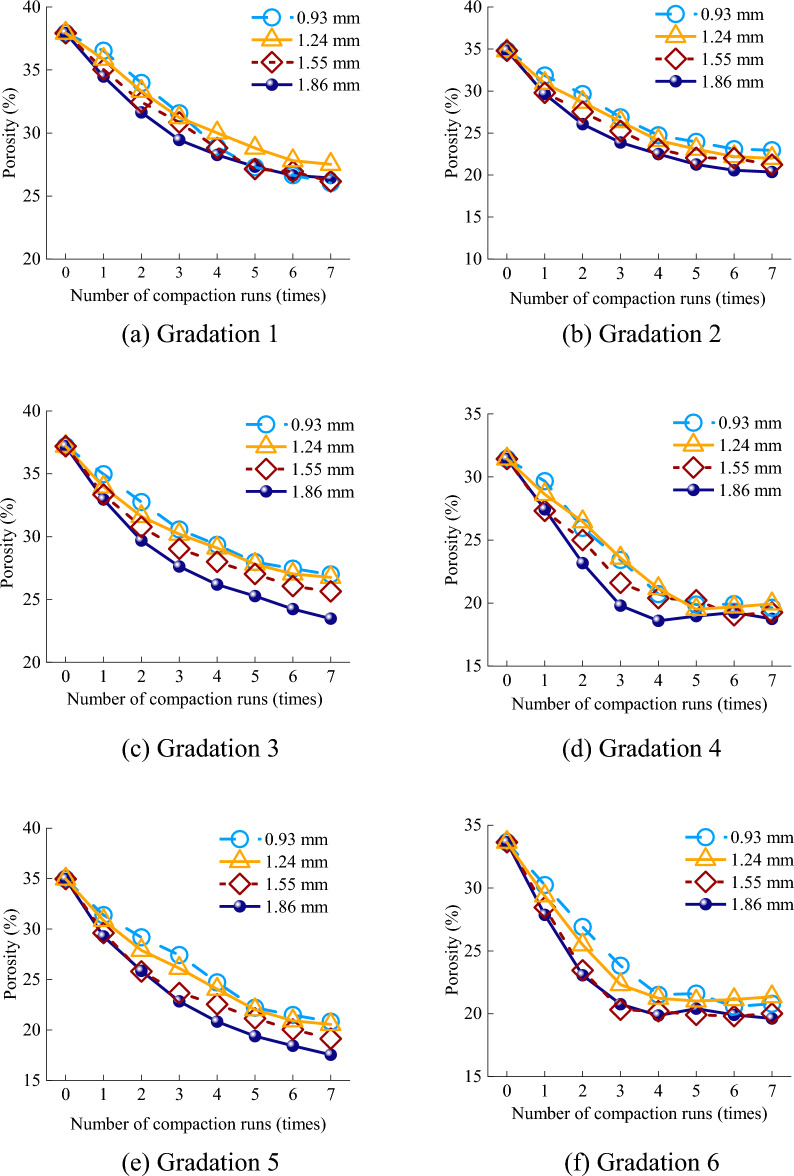
Effect of vibration amplitude on the porosity.

As previously mentioned, the vibration amplitude in the simulation can be changed by adjusting the weight of the eccentric block. In Recurdyn, the eccentric block quality can be adjusted by changing the mass to “user input” on the eccentric block properties page, and the driving function of the eccentric block can be adjusted according to Eq. (3) (the excitation force). The simulation results for the influence of different amplitudes on the porosity at 33 Hz are shown in Fig. 9.

As shown in Fig. 9, as the small rock particles content increased from gradation 1 to gradation 3, the impact of the vibration amplitude on the porosity gradually increased. For gradation 1, the compaction porosities at the amplitudes of 0.93 mm and 1.86 mm were 26.05% and 25.67%, respectively, with a difference of only 0.38%. As the small particle content gradually increased, the compaction porosities of gradation 3 under these two amplitudes were 26.99% and 23.48%, respectively, with a difference of 3.51%. This occurred because the depth of action increased with amplitude, which was conducive to the continual movement of small and medium particles between the rock particles, thereby reducing the porosity. However, the small rock particles of gradations 4 and 6 were dominant, and the large amplitude caused the porosity to rebound. In instances where the small particle content was less than or equal to the large particle content, the optimal amplitude was 1.86 mm. Conversely, when the small particle content surpassed the large particle content, the optimal amplitude was 1.24 mm (Supplementary Information 1).

### Influence of compaction times on settlement amount

**Figure 10:**
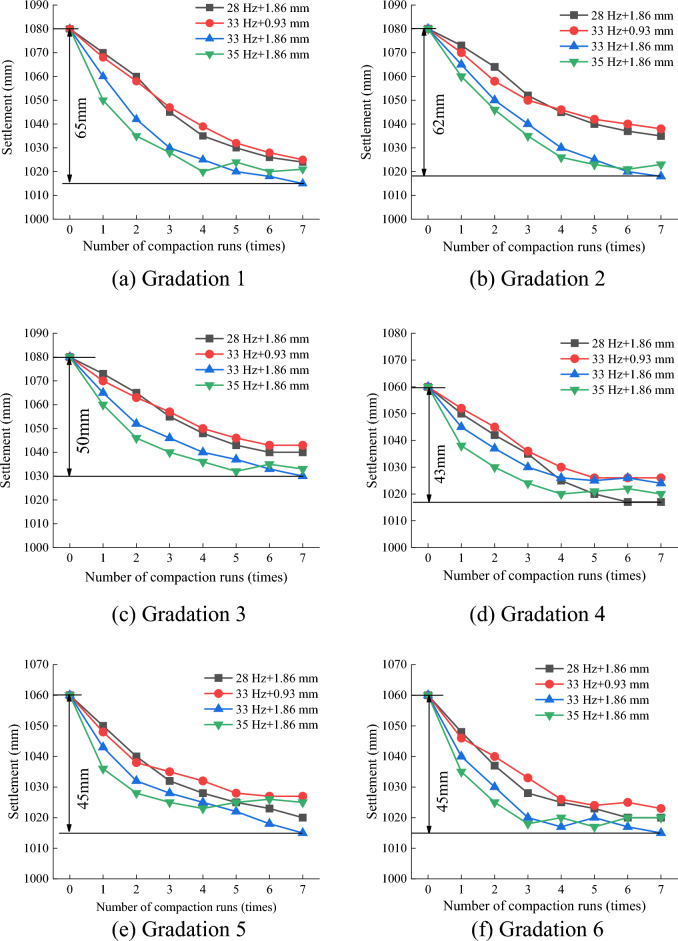
Relationship between the number of compaction passes and settlement.

The longitudinal variation of the center of mass of the vibrating roller was considered the settlement amount, and the relationship between the settlement amount and the number of compactions is shown in Fig. 10.

As shown in Fig. 10, the average settlement amount was approximately 50 mm, and the change in the settlement amount as a function of the number of compaction passes could be divided into three stages, as follows. (1) Rapid growth stage (1–3 compactions). In this stage, the average settling rate for gradation 1 (16.67 mm/pass) was the fastest, and that for gradation 4 (9.67 mm/pass) was the slowest. (2) Slow growth stage (4–5 compactions). The average sedimentation rate for gradation 1 (7.5 mm/pass) was the fastest, and that for gradation 5 (4 mm/pass) was the slowest. The sedimentation rate decreased significantly, and the settlement was more susceptible to rebounding at larger vibration frequencies and amplitudes. (3) Stable stage (six or more compactions). In this stage, the average sedimentation rates for gradations 1, 3, and 5 were all 3 mm/pass, the other gradations were 2 mm/pass, and the settlement amount did not change significantly. Therefore, increasing the number of compaction passes did not have engineering significance. Consequently, in practice, an appropriate number of compaction passes must be selected during the actual compaction process (Supplementary Information 1).

## Mesoscopic response of particles

### Particle displacement field

**Figure 11:**
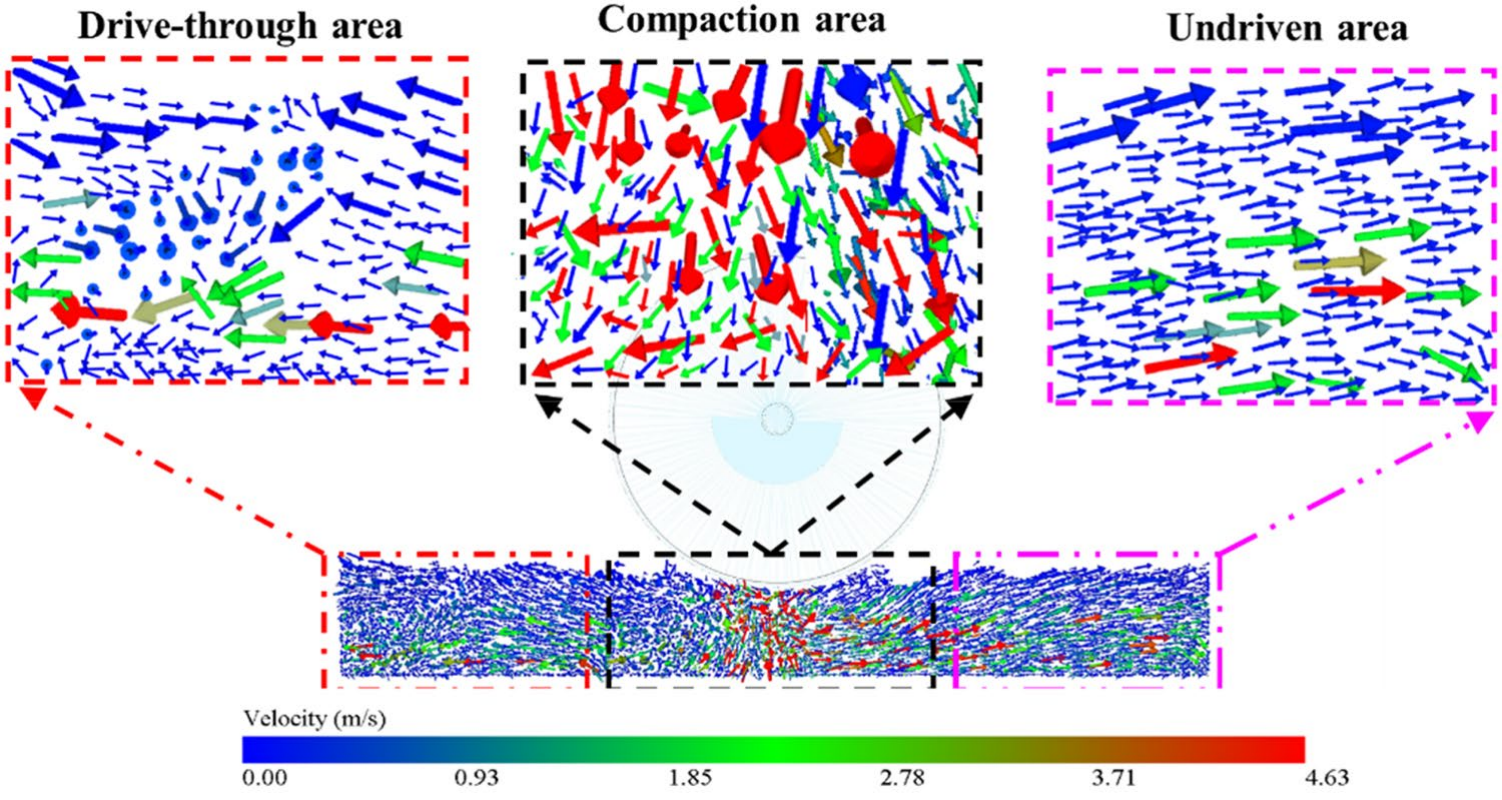
Displacement field of particles under vibrating compaction.

In EDEM, the directions of particle movements are presented in the form of displacement vectors. The compaction effects, as displayed in EDEM, are shown in Fig. 11.

As shown in Fig. 11, when the roller rolled from left to right, the particles in the drive-through area moved to the left and downward as a result of the extrusion of the particles in the compaction area. In the compaction area, the upper particles moved downward, and the particles in the middle and lower layers moved both downward and sideways in response to the action of the vibratory roller. Furthermore, the particles in the undriven area moved to the right because of the roller motion and particle extrusion. The particles at the boundary moved in the opposite direction owing to the boundary constraint, and the particle velocity was highest in the compaction area and in the middle of the other two areas. The main reason for this was that the particles exhibited a large vertical displacement due to the vibration, compressing the space between the particles and making the material denser. The continuous compactions caused the vertical displacement of the particles to diminish over time; thus, the lateral displacement of the particles eventually stabilized.

### Effect of compaction parameters on particle motion

**Figure 12:**
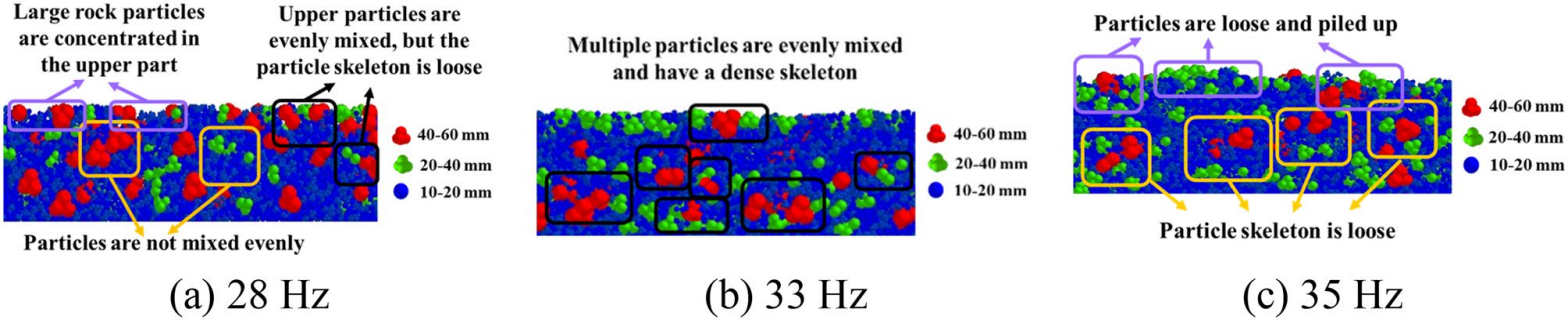
Compaction cross-section diagrams under different vibration frequencies.

Figure 12 shows the final compaction sections at different vibration frequencies for gradation 5. At 28 Hz, the particle mixing was nonuniform and the particles were less interlocked. Large rock particles were concentrated in the middle and upper parts of the mixture, as shown in Fig. 12a. As shown in Fig. 12b, the mixing and distribution uniformity of the rock particles, as well as the interlocking and interparticle filling at 33 Hz, were the best, and the surface was smoother. At 35 Hz, the particle skeleton was destroyed, large and medium rock particles were dispersed among the small rocks, and a large number of loose small and medium particles accumulated in the upper part of the mixture, as shown in Fig. 12c. The reason for this phenomenon was that the skeleton structure between large rock particles was destroyed in the later stages of compaction, and the small and medium rock particles could not maintain a stable position, resulting in particle stratification.

**Figure 13:**
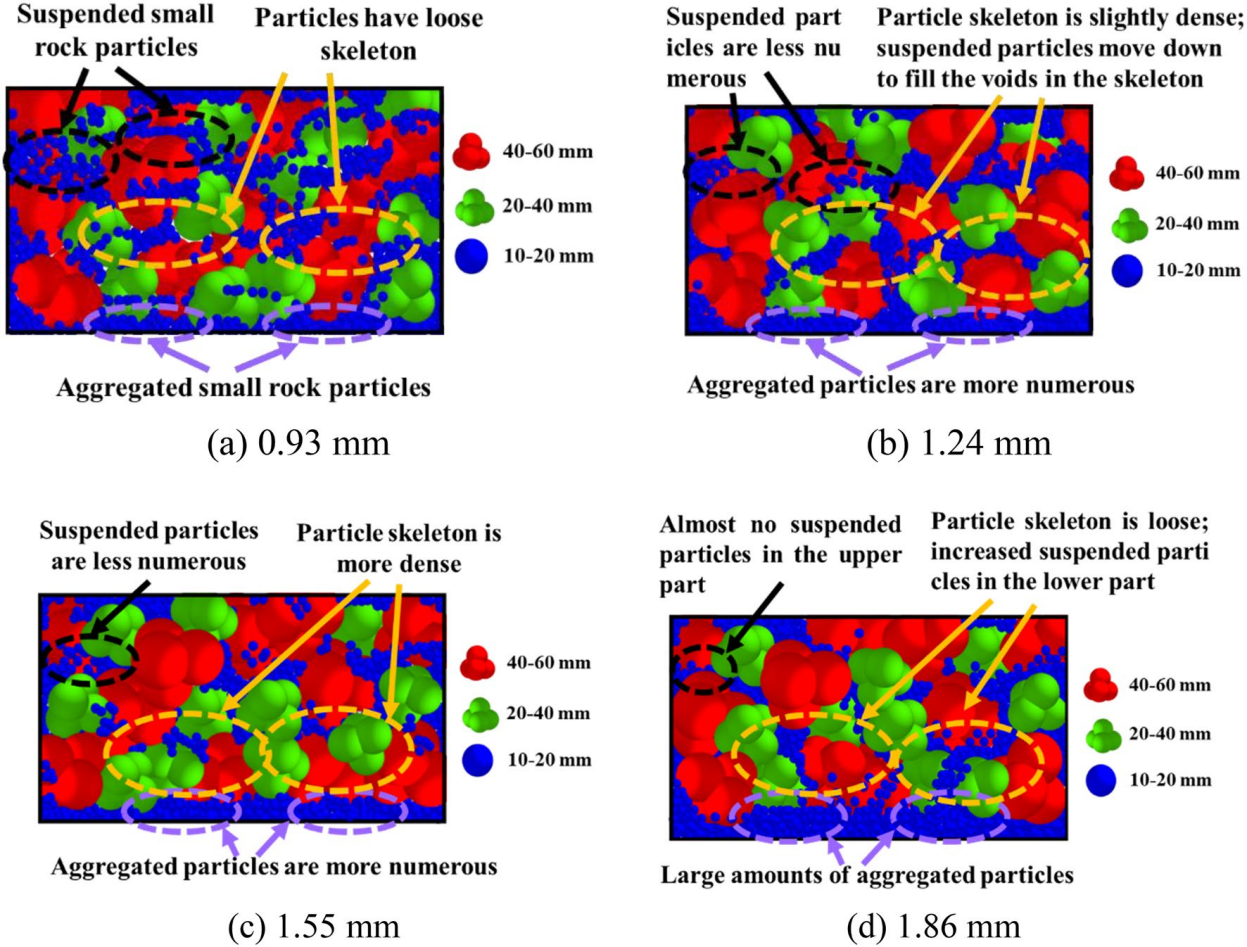
Compaction cross-sections under different vibration amplitudes.

The compaction sections of gradation 5 at different amplitudes are shown in Fig. 13. Figure 13a shows the smallest depth of action; therefore, the lower particle skeleton is looser. As the amplitude increased, the smaller particles became packed together more closely, increasing the contact area between the particles and resulting in a denser skeleton. Furthermore, the number of small rock particles suspended in the rock skeleton decreased, and they filled the gaps in the skeleton or moved to the lower part of the mixture, as shown in Fig. 13b,c. As shown in Fig. 13d, an excessive amplitude of 1.86 produced the skeleton, and the small particles in the lower part could not maintain a stable position and moved upward through the rock particles. A comparison between Figs. 12 and 13 revealed that both the vibration frequency and amplitude affected the movement and arrangement of the particles, and the impact of the vibration frequency was greater than that of the amplitude.

### Contact force and displacement between particles

#### Forces on rock particles of various gradations

**Figure 14:**
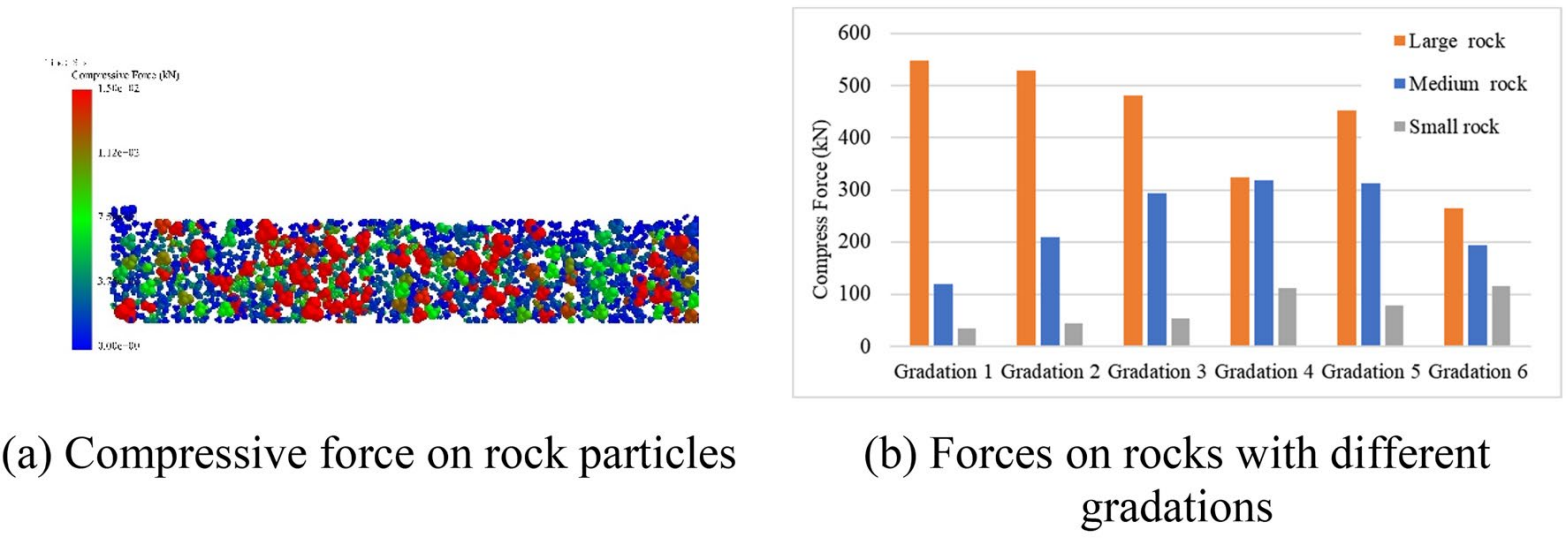
Compressive force on particles.

In EDEM, a “slice” section with a normal vector (1,0,0) was established, and it was used to display the compressive force of the particles, which is shown in Fig. 14. As shown in Fig. 14a, the stress on the rock particles decreased from the middle compaction area to both sides.

The stress on the particles of different sizes at the middle position of each gradation after the fourth pass of compaction is shown in Fig. 14b. The content of large rock particles from gradations 1 to 4 gradually decreased, but the compressive force of the large rock particles did not change significantly. Compared to gradation 1, the stress on the large rock particles in gradations 2 and 3 decreased by 3.6% and 12.2%, respectively. However, for the same gradations, the compressive force on the medium rock particles increased by 73.5% and 145.0%, respectively, whereas that on the small rock particles only increased by 29.0% and 54.8%, respectively. However, the medium rock particles could not replace the dominant force-bearing positions of the large rock particles. For example, in gradation 4, when the content of medium rock particles was 20% greater than that of large rock particles, the stress on the large rock particles was still 2.2% higher than that on the medium rock particles. Similarly, for gradation 6, the content of small rock particles was the largest, but its compressive force was only 56.8% that of the large rock particles. Therefore, for each gradation, the larger the particle size, the greater the force.

#### Force and displacement of particles under different compaction passes

**Figure 15:**
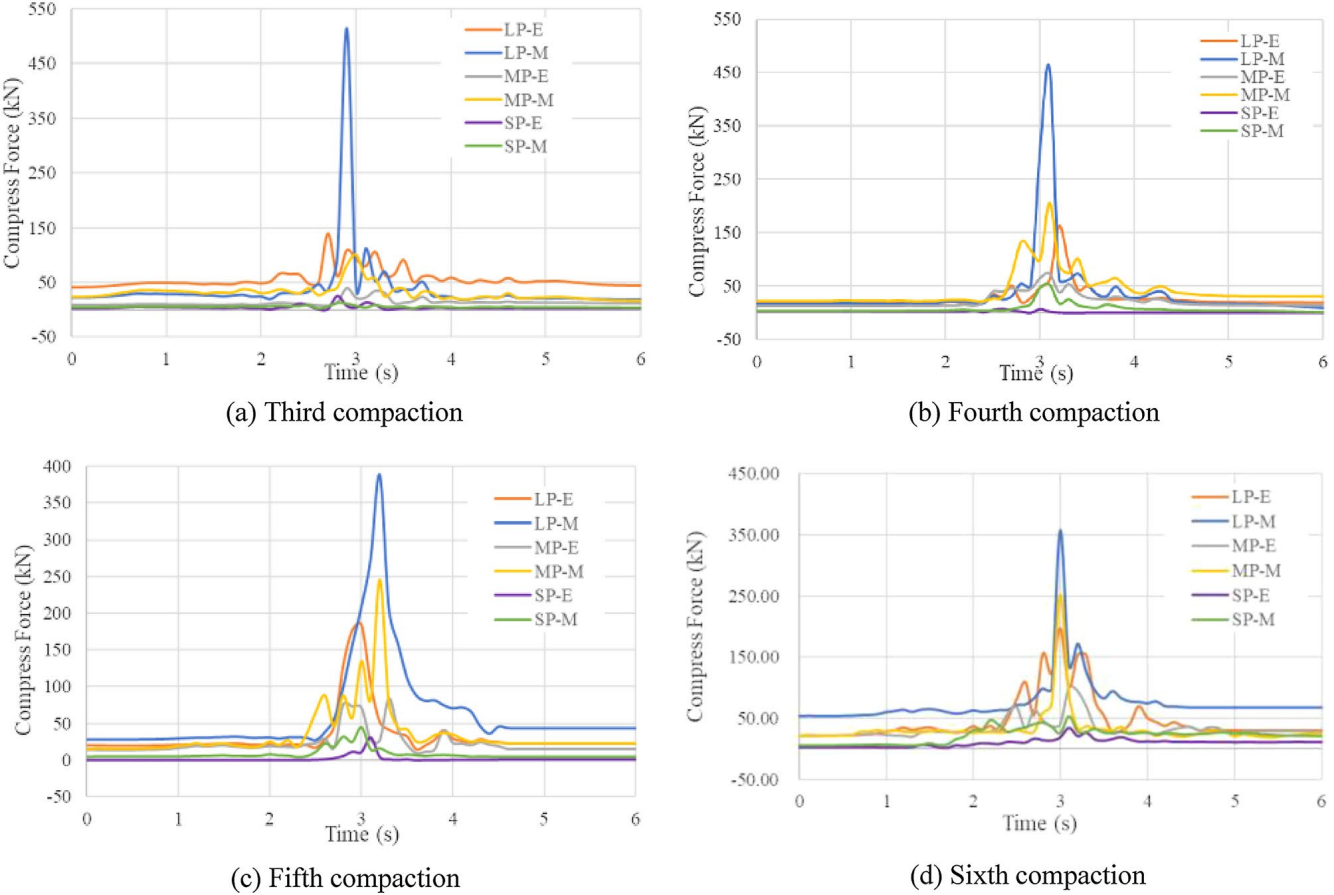
Compressive force on particles for different compaction passes (E means edge position; M means middle position).

**Figure 16:**
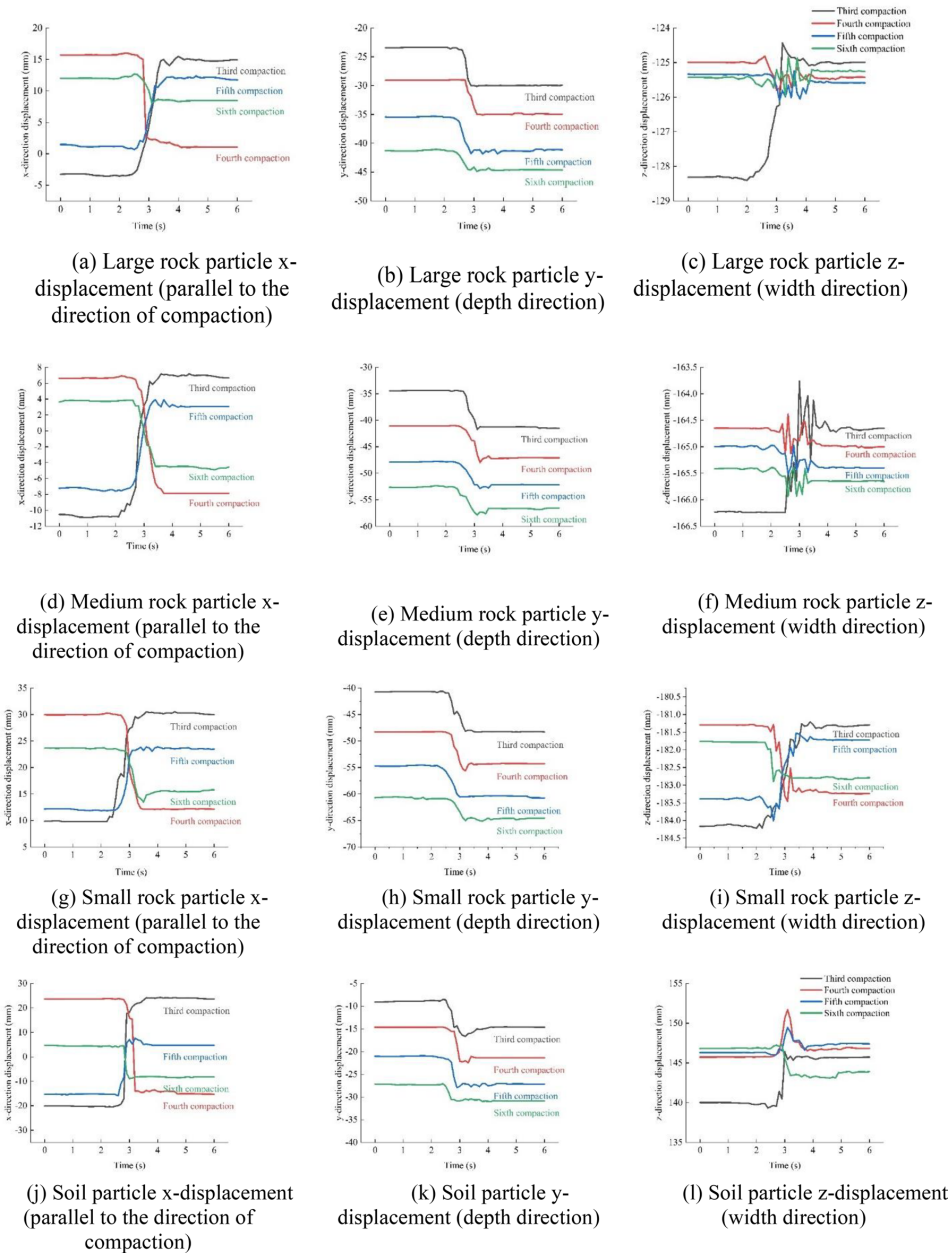
Displacement of each particle in the middle position.

**Figure 17:**
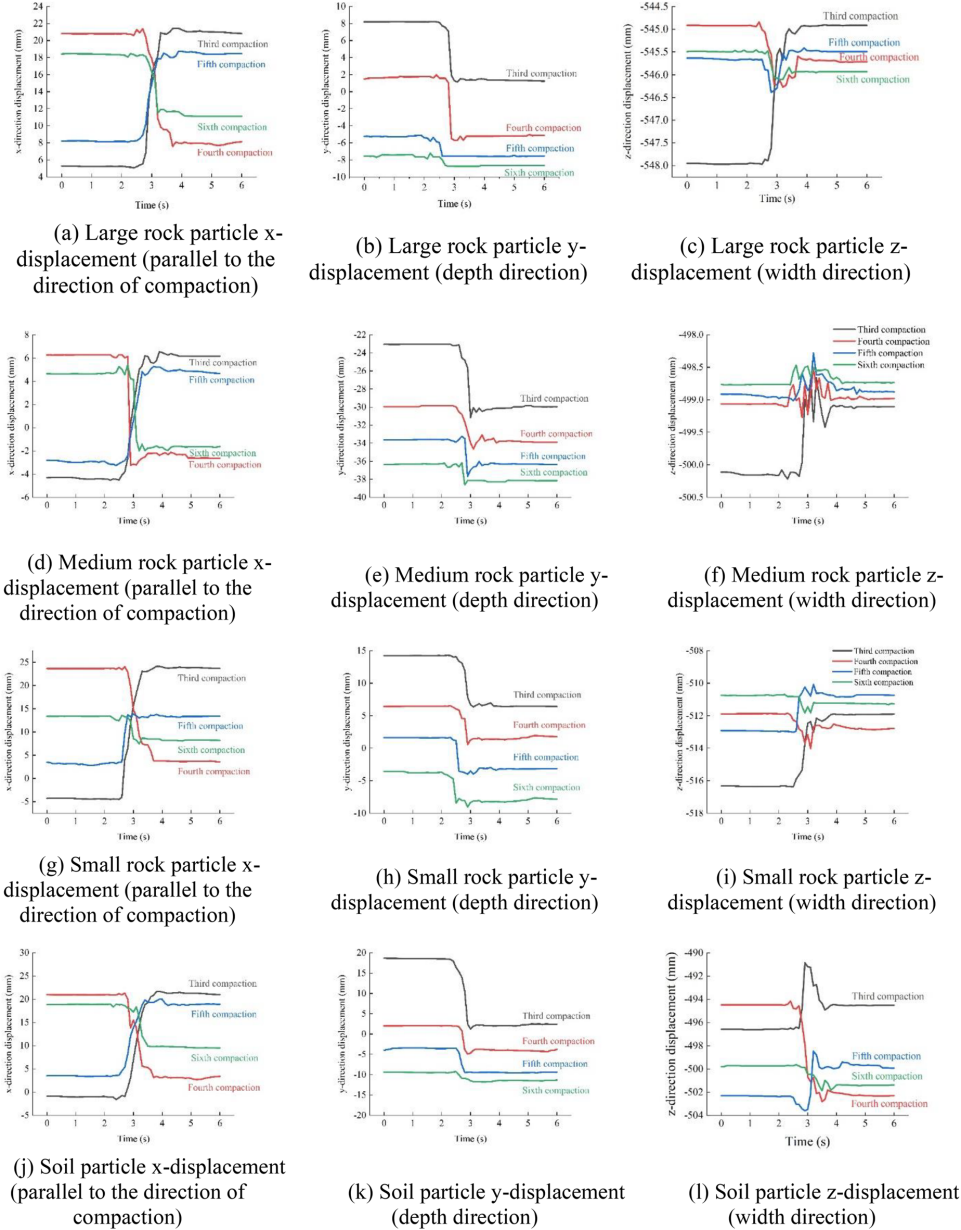
Displacement of each particle at the edge position on both sides.

Taking into account the stability of the data, an analysis was conducted on the force exerted on and the displacement of the particles during the third to sixth compaction passes. The forces acting on particles of various sizes at the middle and edge positions under different compaction passes are shown in Fig. 15, and the displacement changes are shown in Figs. 16 and 17 (Supplementary Information 1). The selected particles had approximately the same heights and were always the same. As shown in Fig. 15, for a given compaction pass, a larger particle size resulted in a greater force. Moreover, for particles of the same size, the force at the middle position was greater than that at the edge position, which is consistent with Fig. 14a.

As seen in Figs. 16 and 17, there was no obvious particle displacement, but slight longitudinal (x-direction) motion occurred owing to the movement of particles in the nearby compaction area when the vibratory roller was not driven to this area in the early stage. Large vertical (y-direction) and longitudinal (x-direction) movements are visible when the vibratory roller was driven to this area. After this, the vibratory roller left this area, the particles bounced slightly upward, and the lateral (z-direction) displacement of the particles repeatedly fluctuated.

For the same cross section, the changes in the vertical, longitudinal, and lateral displacement of the particles under different compaction passes are as follows.

1. Vertical displacement. In the third compaction pass, the vertical displacement of all the rock particles was about 7 mm. During the fourth to fifth compaction passes, the displacement of large and medium rock particles was about 5 mm, and the displacement of other particles remained at about 7 mm. In the sixth compaction pass, the displacement of the large and medium rock particles was approximately 3 mm, and the displacement of the small rock particles was approximately 4 mm.
2. Lateral displacement. During the third compaction pass, the displacement of all the particles was about 3 mm. During the fourth and fifth compaction passes, the displacement of the large and medium rock particles was approximately 0.4 mm, and the displacement of the small rock particles was approximately 1.5 mm. During the sixth compaction pass, the displacement of both the large and medium rock particles was approximately 0.2 mm, whereas the displacement of the small rock particles was 1 mm.
3. Longitudinal displacement. During the third compaction pass, the displacement of all the particles was about 18 mm. During the fourth and fifth compaction passes, the displacement of the large and medium rock particles was approximately 12 mm, and that of the small particles through the rock particles was approximately 14 mm. During the sixth compaction pass, the displacement of the large and medium rock particles was approximately 5 mm, and that of the small rock particles was approximately 8 mm.

The analysis revealed that the displacement in all directions showed a trend of rapid growth, slow growth, and stability. This was combined with an analysis of the stress on the particles (Fig. 15) and settlement changes (Fig. 10) in different compaction passes. In the first to third compaction passes, the large particles received the greatest force, causing large displacements and driving the small and medium particles to move. When compacted four to five times, the mixture gradually became denser, and the small and medium particles had more contact, resulting in a reduction in stress on the large rock particles and an increase in stress on the other particles. At this time, the positions of the large rock particles were stable, but small displacements were caused by the small particles. During the sixth compaction pass, the density of the mixture stabilized, and the force on the particles tended to be stable. Therefore, the particle movements were minimal, and the corresponding settlement amount ceased to increase. A comparison between Figs. 15 and 17 shows that the force on the particles at the edge was less than that in the middle; this resulted in a smaller vertical displacement of the particles at the edge than that in the middle. However, because of the motion of the particles in the middle, the lateral and longitudinal displacements of the particles at the edge were larger than those in the middle (Supplementary Information 1).

### Particle rotation

**Figure 18:**
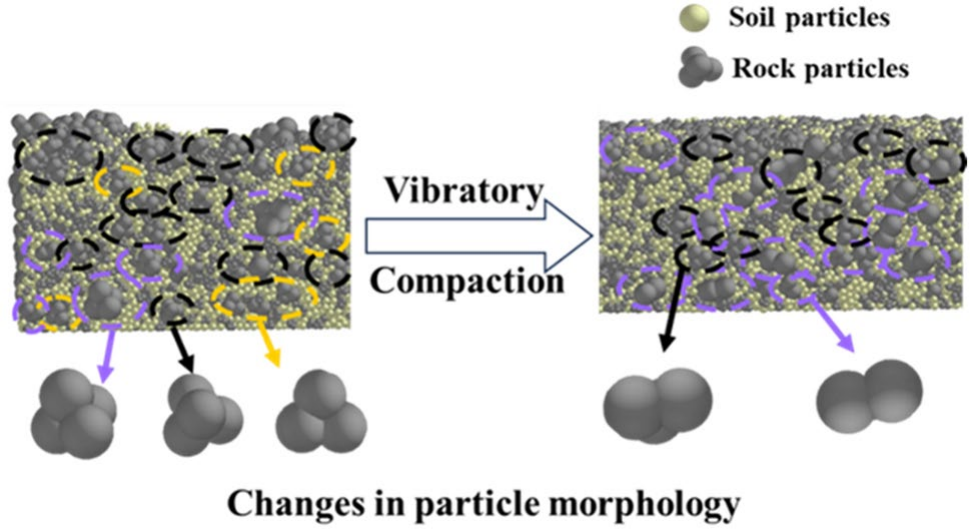
Changes in the particle structure as a result of the compaction.

Figure 18 shows the changes in the particles in the longitudinal section owing to the vibration compaction (Supplementary Information 1). Both the position and morphology of the particles changed, and in the process of vibration compaction, the particles in the slag not only exhibited vertical, horizontal, and lateral motion, but also rotational motion. The long axes of the particles gradually aligned with the direction of the compaction of the vibration roller.

**Figure 19:**
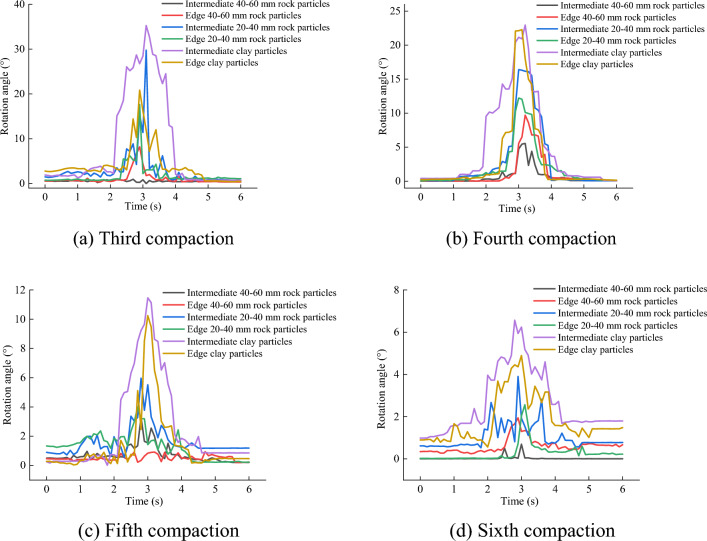
Particle rotation angles.

The particles in the middle and on the edges were selected, and their rotation angles were obtained by multiplying the cumulative time steps by the angular velocity of the particles^35^. The rotation angle exhibited by each particle as the result of the vibration compaction is shown in Fig. 19. The larger the rock particle size, the smaller the rotation angle. The maximum rotation angle of the rock particles was approximately 16°, and the rotation angle decreased gradually as more compaction time elapsed. The rotation of the soil particles usually preceded that of the rock particles, and their rotation angles were larger than those of the rock particles. The results show that the particles rotated during the process of vibration compaction. When the re-chimerism between the rock particles increased their friction, the rock particles no longer rotated significantly. In addition, the soil particles were more affected by the vibration, and their rotation angles were larger than those of the rock particles.

Discrete element software was used to clearly observe the motion of and forces on the packing particles as a result of the vibration compaction of the slag. In the process of vibration compaction, the particles collided with each other. Consequently, the particles rotated and their relative positions changed. This rearrangement reduced the porosity, which achieved the desired compaction.

## Further experimental results and analysis

### Settlement and displacement

**Figure 20:**
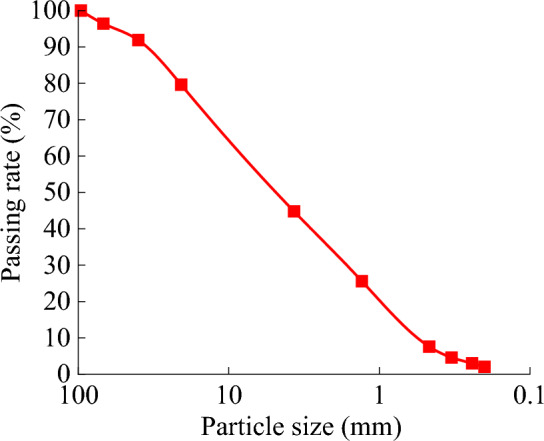
Gradation curve of soil-rock mixture obtained from the field.

A field compaction test of a soil-rock mixed roadbed was conducted in the K76 + 648 to K76 + 863 sections of the Jihe Expressway reconstruction and expansion project in Shenzhen, Guangdong Province, China. The roadbed thickness was 25 cm. The particle gradation curve, obtained through field sampling and screening, is shown in Fig. 20. It resembles the simulation of gradation 4.

**Figure 21:**
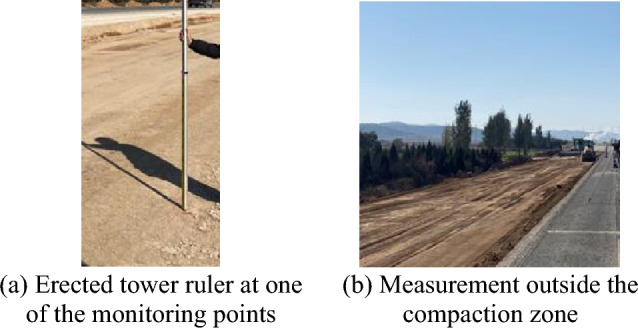
Site of settlement measurements.

**Figure 22:**
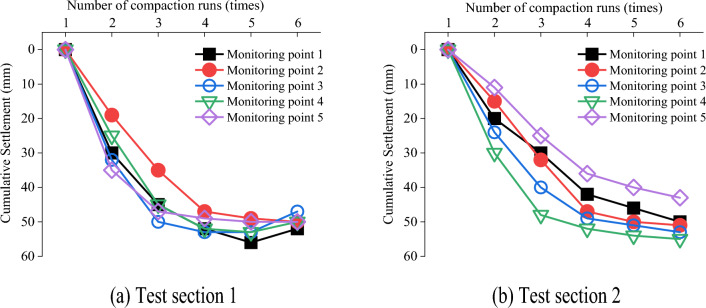
Changes in the settlement as a function of compaction passes for each monitoring point.

Along the compacted road section, five monitoring points were established at equal intervals. In addition, a fixed point was selected on the outside of the road section, and the starting elevation of each monitoring point was measured. After each compaction, the elevations of these monitoring points were measured again, and the differences in elevation were considered settlement differences. The layout of the measurement sites is shown in Fig. 21. Two sets of tests were conducted, the results of which are shown in Fig. 22 (Supplementary Information 1).

Figure 22 indicates that the settlement first exhibited a rapid decline, then a slow decline, and then stability. In Fig. 22a, the settlement of monitoring points 1, 3, and 4 rebounded during the fifth and sixth compaction passes, indicating a high pressure point. The average settlement at each monitoring point in test section 1 was 49.8 mm, and that in test section 2 was 50.4 mm. The simulated settlement of gradation 4 was 43 mm, which was similar to the settlements measured in the field. Figures 10 and 22 demonstrate that the simulated settlement trends were consistent with the field test results.

**Figure 23:**
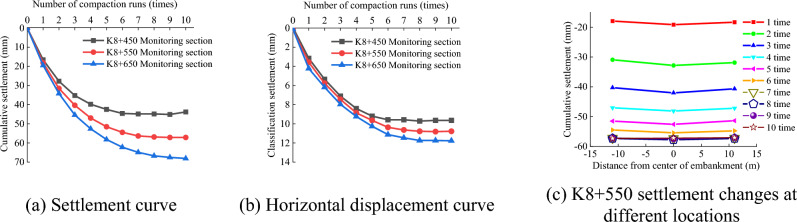
Settlement curves for sections K8 + 450, K8 + 550, and K8 + 650.

Hu et al.^36^ conducted a field compaction test on a highway project by burying monitoring elements in a soil-rock concrete subgrade with different thicknesses of loose paving. The settlement and horizontal displacement of three monitoring points in three different sections (K8 + 450, K8 + 550, and K8 + 650), as well as the settlement amount of the K8 + 550 section at different distances from the center of the roadbed, were measured. The bulk thicknesses of K8 + 450, K8 + 550, and K8 + 650 were 40, 60, and 80 cm, respectively. The test results are presented in Fig. 23.

Figure 23a,b indicate that the settlement curves for different thicknesses of loose paving exhibit a trend characterized by an initial increase, followed by stabilization, and finally reaching a constant value. The change in the horizontal displacement was the same as that in the vertical displacement, but the settlement amount was much smaller than that of the vertical displacement. As shown in Fig. 23c, the settlement at the center of the roadbed was significantly greater than that on both sides. The field test and simulation results were consistent with the test results of Hu et al.^36^.

### particle rotation comparison

A few studies have been conducted on particle rotation during the compaction of soil-rock mixtures. For example, Xie et al.^37^ used a self-developed large-scale vibration compaction instrument combined with X-ray computed tomography (X-CT) to scan graded gravel, and analyzed the dynamic evolution process of particles. They found that, as compaction proceeded, the long axes of the particles gradually tended to be distributed horizontally. These results are consistent with those reported by Xie et al.^37^

## Analysis and discussion of results

A comparison between the simulation and field test results indicated that the slag particles in the early stage of compaction primarily moved vertically, and the particle compression caused by the vibratory roller decreased the porosity. With continuous compaction, it was difficult for the particles to continue to undergo vertical motion compression; instead, the particles underwent lateral displacement and rotation to reduce the gaps between the particles and maintain the compaction. The displacement and rotation of the particles in the mesoscope gradually decreased and eventually stabilized, and the granular chimera formed by the particles became a stable skeleton structure. The porosity decreased, and the particles became compacted and stable. Therefore, the process of vibration compaction is a process in which particles constantly move to adjust their positions and maintain stability.

## Conclusion

In this study, the discrete element method was used to simulate the vibration compaction of slag with different gradations. The particle compaction, particle movement, and compressive force resulting from the vibration compaction were measured. The following conclusions were drawn.

1. The analysis revealed that medium rock particles primarily connected large rock particles and enhanced their overall stability. The vibration frequency and amplitude of the mixture were mainly affected by the relationship between the small and large rock particle content. When the small rock particle content was less than or equal to the large rock particle content, the optimal amplitude was 1.86 mm and the optimal vibration frequency was 33 Hz. Otherwise, the optimal amplitude was 1.24 mm and the optimal frequency was 28 Hz.
2. The vibration frequency and amplitude affected the movement and arrangement of the particles. Small vibration frequencies resulted in a nonuniform particle distribution and a weak skeleton structure; however, excessive vibration frequencies led to particle delamination. The larger the amplitude, the denser the skeleton, and the more the small rock particles accumulated at the bottom of the mixture. An excessive amplitude caused the bottom skeleton to loosen and the small rock particles to rebound upward.
3. The real-time displacement in the first, second, and third compaction passes was caused by the movement of small rock particles driven by large rock particles, and the settlement was approximately 7 mm/pass. Displacement in the later stage of compaction was caused by the movement of large rock particles driven by small rock particles. The settlement during the fourth and fifth compaction passes was approximately 5 mm/pass, that at the sixth pass was approximately 3 mm/pass, and the settlement amount gradually approached zero.
4. During the vibration compaction process, the particles rotated, and the maximum rotation angle of the rock particles was approximately 16°. The rotation angle decreased from the middle to both sides, and the rotation of the soil particles preceded that of the rock particles.

### Supplementary Information

Supplementary Information.

## Data Availability

All data generated or analyzed during this study are include this published article and its supplementary information files.
